# Innate Immune Responses after Airway Epithelial Stimulation with *Mycobacterium bovis* Bacille-Calmette Guérin

**DOI:** 10.1371/journal.pone.0164431

**Published:** 2016-10-10

**Authors:** Erik Tenland, Gisela Håkansson, Nader Alaridah, Nataliya Lutay, Anna Rönnholm, Oskar Hallgren, Gunilla Westergren-Thorsson, Gabriela Godaly

**Affiliations:** 1 Department of Laboratory Medicine, Division of Microbiology, Immunology and Glycobiology, Lund University, Lund, Sweden; 2 Department of Translational Medicine, Division of Clinical Microbiology, Lund University, Lund, Sweden; 3 Department of Clinical Sciences, Division of Respiratory Medicine and Allergology, Lund University, Lund, Sweden; 4 Department of Experimental Medical Science, Division of Lung Biology, Lund University, Lund, Sweden; 5 Department of Translational Medicine, Division of Medical Microbiology, Lund University, Lund, Sweden; Animal and Plant Health Agency, UNITED KINGDOM

## Abstract

*Mycobacterium bovis* bacilli Calmette-Guerin (BCG) is used as a benchmark to compare the immunogenicity of new vaccines against tuberculosis. This live vaccine is administered intradermal, but several new studies show that changing the route to mucosal immunisation represents an improved strategy. We analysed the immunomodulatory functions of BCG on human neutrophils and primary airway epithelial cells (AECs), as the early events of mucosal immune activation are unclear. Neutrophils and the primary epithelial cells were found to express the IL-17A receptor subunit IL-17RA, while the expression of IL-17RE was only observed on epithelial cells. BCG stimulation specifically reduced neutrophil IL-17RA and epithelial IL-17RE expression. BCG induced neutrophil extracellular traps (NETs), but did not have an effect on apoptosis as measured by transcription factor forkhead box O3 (FOXO3). BCG stimulation of AECs induced CXCL8 secretion and neutrophil endothelial passage towards infected epithelia. Infected epithelial cells and neutrophils were not found to be a source of IL-17 cytokines or the interstitial collagenase MMP-1. However, the addition of IFNγ or IL-17A to BCG stimulated primary epithelial cells increased epithelial IL-6 secretion, while the presence of IFNγ reduced neutrophil recruitment. Using our model of mucosal infection we revealed that BCG induces selective mucosal innate immune responses that could lead to induction of vaccine-mediated protection of the lung.

## Introduction

Despite the availability of the BCG vaccine, *Mycobacterium tuberculosis* (Mtb) kills 1.5 million people every year. Intradermal administered BCG vaccine offers limited protective immunity in small children, but largely fails to protect adults from pulmonary tuberculosis (TB) [1]. Several modern immunisation strategies, such as the addition of boosters to improve the current vaccine against TB, are currently tested [2, 3]. Changing the route of administration is another promising avenue. Since BCG is a live attenuated vaccine, it gives rise to a local infection and immune activation at the site of administration. The immunity induced at the site of bacteria entry has been shown to determine the level of protection against several mucosal pathogens [4, 5]. In addition, there is emerging evidence supporting the innate immune memory such as the reprogramming of AECs [6–8]. Mucosal vaccines could potentially provide superior protection against tuberculosis when compared with intradermal vaccination [9].

The lung epithelium is often regarded as passive gas exchange barrier, but it is increasingly evident that these cells possess intrinsic antimicrobial capacity [10, 11]. With an array of Toll-like receptors (TLR), epithelial cells can act as sentinels by inducing innate immune responses and the associated onset of the adaptive immune responses. Epithelial recognition of Mtb by TLR-2, TLR-4, TLR-6, and TLR-9 induces the production of chemokines, cytokines and interstitial collagenase, such as the matrix metallopeptidase-1 (MMP-1) that drives tissue destruction in TB [12–16]. In the lung, neutrophils are a large contributor of the innate immune response and are well known for their ability to phagocyte and eradicate bacteria [17]. Neutrophils contribute to protection during early infection, but also produce high levels of pro-inflammatory cytokines, reactive oxygen species and lytic enzymes. Furthermore, these cells possess the ability to form neutrophil extracellular traps (NETs), which all could exacerbate the pathology [18]. Neutrophils have been demonstrated to migrate to the lymph nodes after infections or vaccination, and were recently shown to be the major cells controlling the spread of T cell responses to distal lymph nodes after immunization [19–23].

The chemokine CXCL8 (IL-8) and the cytokine IL-6 are produced within hours from stimulated AECs [16]. CXCL8 recruits neutrophils to the lung tissue and is later on supported by IL-17 family of cytokines in this process. IL-17A is important in the establishment of protective immunity after TB vaccination and is produced by the CD4^+^ T_H_17 cells during acute and chronic lung disease [24, 25]. Recently, IL-17C and A respectively, were shown to be produced by colon epithelial cells and to mediate mucosal vaccine-induced immunity against tuberculosis [26, 27]. The multi-potent cytokine IL-6 acts in both pro- and anti-inflammatory ways [28]. Exerting its pro-inflammatory qualities, IL-6 suppresses T_reg_ development and favours the differentiation of effector T_H_17 cells during inflammation or infection [28]. Another pro-inflammatory cytokine, IFN-γ, has classically been considered the crucial for disease control and is an important tool in TB diagnosis and vaccination success [29]. Both neutrophils and AECs produce IFN-γ and express the IFN-γ receptor to activate pro- and anti-inflammatory pathways [30]. Activation of these signalling cascades could lead to early protective immunity against TB [31].

We identified the need to further clarify the role of AECs and neutrophils as these cell types are now recognized as active participants of the mucosal immune response. The aim of the current study was to use a BCG infection model to illustrate the initial events during mucosal immunity in the lungs. In this study, we investigated BCG activation of neutrophils and AECs from the production of cytokines and receptor expression, to functional recruitment of neutrophils and NET production. We could reveal that BCG affected neutrophil function and altered the expression of epithelial IL-17 receptors. The presence of IL-17/IFN-γ exaggerated epithelial IL-6 secretion and BCG was found to induce NETs. We thus analysed the beneficial effect of mucosal vaccination as well as possible pathological event of using live airway bacteria for the induction of protective immunity in the lungs.

## Materials and Methods

### Ethical Statement

The Swedish Research Ethical Committee in Lund (FEK 413/ 2008) approved the isolation of the bronchial material for primary cell cultures. Bronchial material for primary cell cultures was obtained from lung explant from healthy donors with irreversible brain damage and with no history of lung disease. Lungs were to be used for transplantation but could instead be included in this study as no matched recipients were available at that moment. Written consent was obtained from their closest relatives, in accordance to the Declaration of Helsinki.

### Cell culture

Primary bronchial epithelial cells were obtained from lung explant from donors with no history of lung disease. Bronchial tissue was dissected from lungs and kept in DMEM supplemented with gentamicin, PEST, Fungizone and 10% FBS (all from Gibco, Paisley, UK) until further isolation. After removing intraluminal mucus and surrounding tissue, bronchi were digested in 0.1% Protease (Sigma, St. Louis, MO, USA) prepared in S-MEM supplemented with gentamicin, PEST and Fungizone for 24 h. Human bronchial epithelial cells (HBEC) were recovered by repeated intraluminar rinsing with DMEM supplemented with gentamicin, PEST, Fungizone and 10% FBS. Cells were filtered through a 100 μm strainer (Falcon, Becton Dickinson, Oxford, UK) and seeded in cell culture flasks coated with 1% Collagen-1 (PureCol, Inamed Biomaterial, Freemont, CA, USA) in BEGM cell culture medium (Lonza, Germany). The following day cells were thoroughly washed with a medium change every other day. Experiments were performed in passage 3.

Primary Human Umbilical Vein Endothelial Cells (HUVEC) (Lonza, Germany) were cultured in endothelial cell growth medium EGM-2 (Lonza, Germany) supplemented with the EGM-2 bullet kit (Hydrocortisone, 0.4% hFGF-B, 0.1% VEGF, 0.1% R3-IGF-1, 0.1% Ascorbic Acid, 0.1% Heparin, 2% FCS, 0.1% hEGFMl and 0.1% Gentamicin/ Amphotericin B (GA- 1000; Lonza, Germany) in 37°C, 5% CO_2_. The cells were used in passages 2–5, according to manufacturers’ instructions.

Human venous blood neutrophils were isolated from healthy volunteers by Polymorphprep density gradient (Axis-Shield, Oslo, Norway) according to manufacturers instructions as described previously [32].

### Bacteria and Infection

*Mycobacterium bovis* BCG Montreal strain containing the pSMT1 shuttle plasmid was prepared as previously described [33]. Briefly, the mycobacteria were grown in Middlebrook 7H9 culture medium, supplemented with 10% Albumin/Dextrose/Catalase (ADC; Becton Dickinson, UK) and hygromycin (50 mg/mL, Roche, UK), the culture was dispensed into vials, glycerol was added to a final concentration of 50%, and the vials were frozen at -80°C. Prior to each experiment, a vial was defrosted, added to 10 ml of 7H9/ADC/hygromycin medium, and incubated with shaking for 72 h at 37°C. Mycobacteria were centrifuged for 10 min at 3000 *g*, washed two times and re-suspended in sterile PBS. The bacteria were quantified based on luminescence by adding 0,1% Decanal and measuring relative luminescence units in a luminometer (TrisStar, Berthhold Technologies, Germany). Infection with BCG was performed at low multiplicity of infection (MOI) 1:1 BCG to epithelial cells, HUVEC or neutrophils. We used low MOI to better depict the distribution of current vaccination dose on the large surface area of the airway epithelium. After infection, the cells were incubated at 37°C, 5% CO_2_ for three days (epithelial cells and HUVEC) or 3 hours (neutrophils).

### ELISA

Culture supernatants were collected and kept at -20°C until use. Quantikine ELISAs were performed for the cytokines CXCL8, IL-6, IL-17A, IL-17B, IL-17C, IFN-γ, and for the metalloproteinase pro-MMP-1, as described by the manufacturer (D8000C, D6050, D1700, DY1248, DY1234, DIF50, DMP100, R&D Systems, Denmark). Briefly, human standard and samples were added to a 96-well plate and incubated at room temperature, washed and incubated with conjugate for 2 hours at room temperature. The plate was washed and a substrate was added. Optical density was read at 450 nm with a Tecan Infinite F200 reader (Labsystems Multiskan MS, USA). Sample concentrations were calculated from the standard sigmoid curve.

### Flow Cytometry

Epithelial and neutrophil IL-17RA and IL-17RE expression was investigated by flow cytometry. Cells were infected as mentioned above and stained with primary mouse anti-human IL-17RA antibody (MAB177, 5 μg/ml, RnD Systems, Denmark) or rabbit anti-human IL-17RE antibody (1 μg/ml, ab77527, Abcam, see S1 Fig for optimization). The cells were washed two times in PBS and stained with secondary goat anti-mouse or anti-rabbit IgG (1:300, Alexa Flour 488, Life Technologies, Carlsbad CA, USA) antibody for 30 min on ice. Mouse IgG (5 μg/ml, X0931, Dako, Denmark) or rabbit IgG (1 μg/ml, X0936, Dako, Denmark) were used as isotype controls and unstained cells were used as negative background control. Cells were washed twice in PBS and analyzed by flow cytometry in a FACSCalibur instrument (Becton Dickinson, Oxford, UK). A total of 5000 gated events were collected per sample, and data were analysed by Cytobank software[34]. Median fluorescence intensities (MFI) were calculated and compared to uninfected control or isotype control, whichever was highest.

### Neutrophil migration assay

HUVEC were grown on Transwells inserts (12-well, 3-μm pore size, Corning, NY, USA) in EGM-2 medium, as previously described [16]. As a control, cell viability were analysed by trypan blue exclusion assay according to manufactures instructions (Sigma, St. Louis, MO, USA). A double transwell model system was created by combining primary endothelial cells in the upper insert, and BCG stimulated epithelial cells in the lower compartment. For the cytokine stimulation experiments, recombinant IL-17A and/or IFNγ (10 ng/mL, 7955-IL, 285-IF, RnD Systems, Denmark) were added to the bottom insert 30 min before the addition of BCG.

Leukocyte transmigration assays were performed as previously described [16]. Briefly, neutrophils (1.5×10^6^, 0.5 ml) were added onto the endothelial cells in upper well. After 3 hours, samples were taken from the bottom well for leukocyte counting by Cell Counter (Sysmex, Hamburg, Germany) and analysed for cytokines. Leukocyte migration after addition of recombinant IL-1α or CXCL8 (10 ng/ml, 200-LA, 208-IL, RnD Systems, Denmark) was used as positive controls, while neutralizing CXCL8 antibodies (0.4 μg/ml, MAB208, RnD Systems, Denmark) during BCG infection were used as negative control (S2A and S2B Fig).

### Fluorescence staining of NETs

Isolated peripheral blood neutrophils were seeded on round glass slides (12mm Ø) and allowed to settle for 30 minutes at 37°C in a 5% CO_2_ atmosphere. Bacteria were added to the neutrophils at MOI 1:1 and incubated for 1, 2 or 3 hours followed by fixation in 4% paraformaldehyde. The glass slides were washed two times in PBS and mounted overnight with ProLong® Gold antifade reagent with DAPI (Life Technologies, Carlsbad CA, USA). Samples were studied in Fluorescence Microscope (Scope A.1, Carl Zeiss Microscopy, LLC, USA).

### Picogreen assay

Neutrophils were seeded on plate and allowed to settle for 1 hour at 37°C, 5% CO_2_, followed by incubation with BCG (MOI 1:1) for 3 hours. NETs were digested with 0,3 U/ml of micrococcal nuclease (Thermo Scientific, Rockford, USA), 30 min. NET DNA was quantified using Picogreen dsDNA kit (Life Technologies, Carlsbad CA, USA) according to manufacturer’s instructions. Briefly, samples were mixed with Picogreen reagent for 5 min in room temperature followed by measurement in white transparent 96-well plates at excitation 485 nm, emission 530 nm using Tecan Infinite F200 reader (Labsystems Multiskan MS, USA). 25 nM Phorbol-12-myristate 13-acetate (PMA) was used as a positive control for NETosis.

### Detection of apoptosis with Annexin V

Neutrophils were exposed to BCG for 2 hours and analyzed for Annexin V with “FITC Annexin V”–kit (BD Biosciences Pharmingen, Franklin Lakes, NJ, USA) according to manufactures’ instruction. Briefly, cells were incubated with FITC conjugated Annexin V for 15 min and analyzed by flow cytometry (Acuri C6, BD Biosciences). SSC-A/SSC-H and FSC-A/SSC-A was gated to only include single cell neutrophils. Annexin V was measured at excitation/emission 488/520 nm.

### Western Blot

Neutrophils were incubated with BCG for 1, 2 and 3 hours. Cells were centrifuged at 200xg for 10 minutes and lysed in M-PER™ buffer (Thermo Scientific, Rockford, USA). Cell debris was centrifuged at 2000xg. Protein concentration was determined with Protein Pierce assay (Thermo Scientific, Rockford, USA), and absorbance was measured at 660 nm in a Nanodrop (NanoDrop 2000, Thermo Scientific Rockford, USA). Proteins were stored at –20°C. Equal amounts of protein were separated by SDS-PAGE and blotted onto PVDF membranes. Membranes were saturated by 5% Dry Milk (FOXO3a) or 5% BSA (pFOXO3a) in PBS-T (Santa Cruz Biotechnologies, Heidelberg, Germany), incubated overnight with rabbit polyclonal antibodies against human FOXO3a (1:1000, #07–702 Millipore, Billerica, MA, USA) or human pFOXO3a (1:1000, ab31109-100, Abcam). Bound antibodies were detected by HRP-linked goat anti-rabbit IgG (1:4000, 7074S, Cell Signaling, Danvers, USA) using ECL Prime Western Blotting detection reagent (GE Healthcare, Little Chalfont, UK) and GelDoc equipment (Bio-Rad Laboratories, Hercules, CA, USA). Membranes were stripped with Restore Western Blot Stripping Buffer (Pierce, Rockford, IL), blocked and re-probed with polyclonal HRP-conjugated anti-human GAPDH antibodies (1:1000, sc-25778, Santa Cruz Biotechnologies, Heidelberg, Germany) used as loading control. To quantify protein concentrations, band intensity was measured with ImageJ software28 and normalized against GAPDH.

### Statistical analysis

Cytokine secretion, neutrophil migration and NET production were analysed with one way ANOVA followed by multiple comparisons tests with Tukey’s (Cytokines and migration) or Dunett’s correction (NETs). Median fluorescence intensities of receptor expression were compared with Mann-Whitney test. Significance was accepted at *p < 0.05, **p< 0.01, or ***p < 0.001. Data were examined using Prism (version 6.0f, GraphPad).

## Results

### Expression of cytokines and MMP-1 in BCG stimulated epithelium

**Fig 1:**
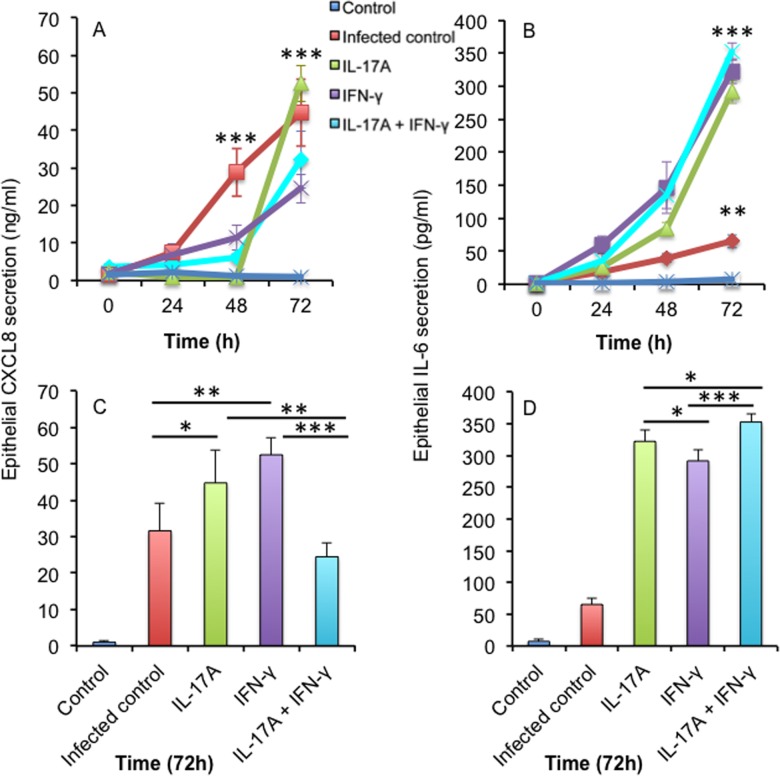
The effect of BCG infection on epithelial cytokine expression. (A) CXCL8 and (B) IL-6 production from epithelial cells stimulated with BCG in combination with IL-17A and/or IFN-γ for 0, 24, 48 and 72 hours, MOI 1:1. The 72 hours time points are in addition represented as bar graphs for (C) CXCL8 and (D) IL-6. The results are depicted as mean ± SD of three experiments. Means were compared with one way ANOVA followed by multiple comparisons test with Tukey’s correction and significance was accepted at *p < 0.05, **p< 0.01, or ***p < 0.001.

To study the cytokine repertoire by BCG stimulated AECs, we measured the cytokines CXCL8, IL-6, IL-17A, IL-17B, IL-17C and IFN-γ. CXCL8 production was found to be dependent on BCG infection (Fig 1A) and the addition of IL-17A significantly increased BCG-induced CXCL8 production after 48 h (p<0.001), while IFN-γ addition peaked epithelial CXCL8 production after 72 h (p<0.001). Increased epithelial IL-6 secretion was induced by BCG stimulation (p<0.01 after 3 h) compared to non-infected control (Fig 1B). The addition of IL-17A and/or IFN-γ had a major impact on BCG induced epithelial IL-6 secretion (p<0.001 after 3 h). We could not detect any IL-17A, IL-17B, IL-17C or IFN-γ secretion from BCG stimulated epithelial cells or neutrophils (data not shown). Production of matrix metalloproteinase from inflammatory sites leads to in alveolar destruction and collagen breakdown [14]. No secretion of MMP-1 was observed from epithelial cells or human neutrophils stimulated with BCG (data not shown).

### IL-17 receptor expression

**Fig 2:**
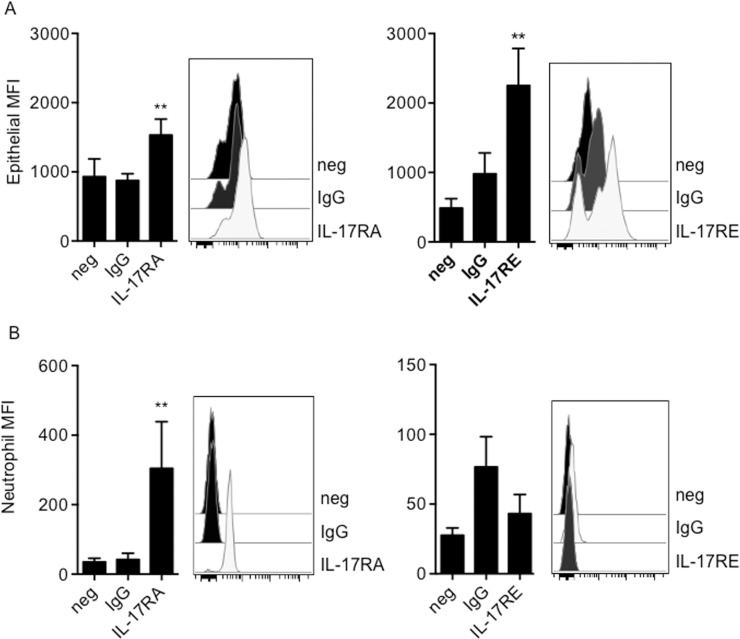
IL-17RA and IL-17RE expression on primary epithelial cells and neutrophils. IL-17RA and IL-17RE expression was assessed by flow cytometry in (A) primary AECs and (B) neutrophils. The results are depicted as mean ± SD of median fluorescence intensity (MFI) of a total of six (epithelial cells) or five (neutrophils) experiments. In addition, representative histograms are shown for each experiment. Abbreviations: secondary antibody control (neg), IgG control (IgG) or receptor expression on cells (IL-17RA/RE). Comparisons were performed with Mann-Whitney test and significance was accepted at *p < 0.05, **p< 0.01, or ***p < 0.001.

It is well established that IL-17 cytokines affect the function of neutrophils. In addition, the cytokine environment determines the reprogramming of AECs. Even though we did not find epithelial or neutrophil production of IL-17, other sources such as T_H_17 cells will be recruited to the site of infection at later stages. We studied the expression of IL-17 receptors on AECs and neutrophils during BCG infection. These heterodimeric receptors all share the common subunit IL-17RA, while specificity is determined by the other subunit [35]. We studied the expression of IL-17RA on primary AECs and primary neutrophils. Cells were stained with anti-IL-17RA antibodies and receptor expression was studied with flow cytometry. Primary AECs had a basal expression of IL-17RA (Fig 2A). Basal expression of IL-17RA was also found on primary human neutrophils (Fig 2B). Since IL-17C has been found to regulate mucosal immunity in the gut, we measured receptor expression of its specific receptor subunit IL-17RE. Epithelial cells were found to have a basal expression of IL-17RE (Fig 2A), while no expression was found on neutrophils (Fig 2B).

Further, we measured the receptor expression of IL-17RA and IL-17RE during BCG infection (Table 1). In contrast to epithelial cells, BCG stimulation of neutrophils significantly decreased IL-17RA expression (p = 0.03). However, infected epithelial cells were found to downregulate the expression of IL-17RE (p = 0.009).

**Table 1:** Median fluorescence intensity (MFI) of studied receptors.

	Epithelial Cells		Neutrophils	
	IL-17RA	IL-17RE	IL-17RA	IL-17RE
Uninfected	1464	2099	369	44*
Infected	1606	1604	123	41*
p-value	p = 0.13	p = 0.009	p = 0.03	p = 0.90

*Values equal to background control

### Neutrophil recruitment

**Fig 3:**
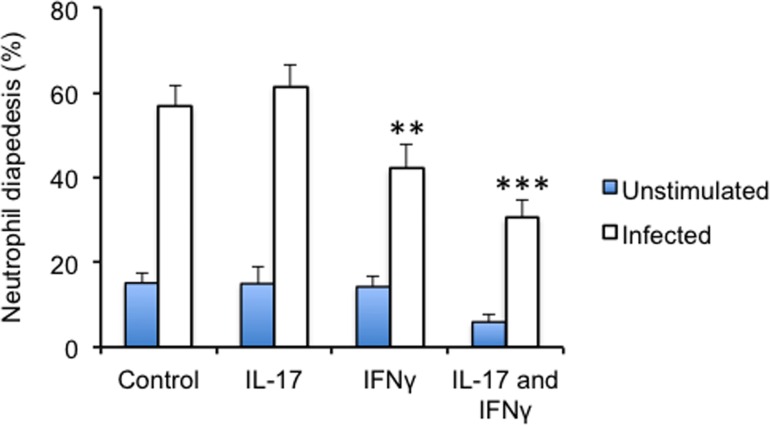
Neutrophil migration during BCG infection. (A) Epithelial cells were seeded to the bottom well of a transwell system and stimulated with BCG for 3 days, MOI 1:1. After infection, primary neutrophils were added to a layer of HUVEC cells in the top insert well. Neutrophil transmigration over the membrane was measured by counting the number of neutrophils in the bottom well compared to the insert after 3 hours. Results are depicted as mean ± SEM percentage of neutrophil diapedesis. Means were compared with students t-test and significance was accepted at *p < 0.05, **p< 0.01, or ***p < 0.001.

The initial interplay between cells during BCG vaccination in the lung is poorly understood. We used a transwell model to study the recruitment of neutrophils during mycobacterial vaccination. Primary epithelial cells were stimulated with BCG for 72 hours in the lower chamber. Uninfected neutrophils were seeded on a layer of primary endothelial cells (HUVEC) in the inserts and the level of neutrophil diapedesis was measured in the lower chamber after three hours (Fig 3A). BCG stimulation of epithelial cells induced significant neutrophil migration compared to non-infected control (p<0.001). BCG infection in the presence of recombinant IL-17A did not increase neutrophil migration further. However, the addition of IFN-γ or IL-17A/IFN-γ combination suppressed neutrophil diapedesis across endothelial cells (p<0.01 and p<0.001 respectively).

### Neutrophil viability

While increased recruitment of neutrophils to the site of infection may be beneficial, we hypothesized that IL-17A signalling also could be involved in neutrophil viability. Increasing the survival of neutrophils could improve the clearance of the pathogen but also pose a risk for pathological damage. We measured the expression of forkhead box O3 (FOXO3), a transcription factor that drives apoptosis through the induction of *Noxa* and *Puma* genes [36]. We could not detect any changes in either FOXO3a or pFOXO3a levels in BCG infected neutrophils at one, two or three hours (S3 Fig). We studied the possible apoptosis process further by using annexin V assay. This second method also did not show any increased apoptosis in infected cells (S4 Fig).

### Neutrophil Extracellular Traps

**Fig 4:**
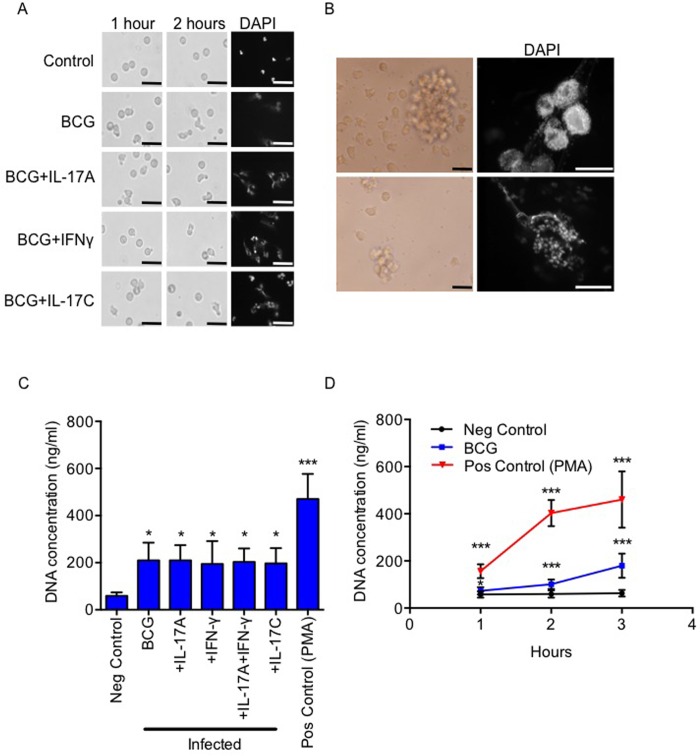
Production of Neutrophil extracellular traps (NETs) during BCG infection. Primary neutrophils were stimulated with BCG for one, two and three hours in the presence of IL-17A and/or IFN-γ. (A) NETs were studied in a light microscope after 2 hours and stained with DAPI and (B) large NET structures were observed in addition to single NETs. (C) Quantification of NETs was performed with Picogreen assay on cell supernatants after 3 hours and (D) over time at 1, 2 and 3 hours. Results are depicted as mean ± SD from a total of 4 donors. Means of samples were were compared with negative control using ANOVA followed by Dunnet’s multiple comparisons test and significance was accepted at *p < 0.05, **p< 0.01, or ***p < 0.001. Bars represent 25 μm (A, B left panel) or 100 μm (B, right panel)

In addition to phagocytosis, it has been suggested that neutrophil use non-phagocytic strategies against mycobacteria [37]. Using microscopy, we found that neutrophils produce neutrophil extracellular traps (NETs) already one hour after BCG stimulation (Fig 4A). NET formation was confirmed by quantification of extracellular DNA (p<0.05, Fig 4B). We found no impact of IL-17A and/or IFN-γ on NET formation. NET production continued and levels increased over three hours (Fig 4C). Interestingly, large (>300 μm) NET formations were frequently observed (Fig 4D).

## Discussion

BCG vaccination relies on mucosal cells to provide the first line response. These initial events are crucial for the formation of effector memory T cells and central memory T cells to mediate the recall responses [38]. One of the initial events is the enhanced BCG-induced apoptosis of mucosal cells that is proposed to be a way to improve vaccine efficacy [39]. BCG was earlier reported to induce macrophage apoptosis by dephosphorylation of transcription factor FOXO3 [36]. In neutrophils, we did not observe any change in FOXO3a levels upon BCG infection, nor could we detect apoptosis in these cells, but we observed that neutrophils formed NETs shortly after encountering mycobacteria. NETosis was recently proposed to differ from apoptosis and necrosis, as the nuclear and granular membranes disintegrate during NETosis, but the plasma integrity is maintained [40]. Interestingly, the NET formations increased with infection time, although we did not find any synergistic effect on NETs in the presence of IL-17 or IFN-γ. During NETosis, viable neutrophils can form NETs that consists of mitochondrial or nuclear DNA, formed in a rapid and non-lytic manner [41, 42]. NETs contain DNA and several biologically active cytosolic and granular proteins that can attract and activate macrophages. Neutrophil cell death leads also to shedding of IL-6R and to progression of the inflammatory process. The less beneficial effects of NETs are linked to negative lung function in patients with chronic lung diseases such as ALI/ARDS and cystic fibrosis [43]. The importance of NET formation is not clear, but these structures could aid in the maintenance of local inflammation. Interestingly, Mtb induced NETs formation was previously proposed to be a first step in its pathogenicity [44, 45]. This similarity could thus be beneficial for mucosal BCG-vaccination.

Addition of IFN-γ to infected AECs increased epithelial IL-6 and CXCL8 secretion, and increased neutrophil diapedesis across endothelial cells. During acute infection, IL-6 is known to suppress mucosal innate immune responses in order to induce acquired immunity. Infiltrating neutrophils produce soluble IL6 receptor (sIL6R), either from proteolytic cleavage of its membrane bound form or by sIL6R released upon neutrophil cell death, which alters mucosal chemokine production to attract monocytes and T lymphocytes [46]. Inflammation in IL-6^−/−^ mice revealed that IL-6 is necessary for T cell recruitment and this cytokine is also pivotal in T cells differentiation towards T_H_2 and T_H_17 [47, 48]. Moreover, IL-6 induces a subset of T helper cells distinct from T_H_1 and T_H_2 cells, namely the formation of IL-17A secreting T_H_17 cells [49]. Locally produced IL-6 could thereby shift the immune responses towards acquired by increasing the formation of CD4^+^ memory T cell.

Neutrophil extravasation into inflamed tissues involves several factors, such as selectins, integrins and several other adhesion molecules and cytokines that tightly regulate this event [50]. We observed that the presence of IFN-γ during BCG infection dampened neutrophil endothelial diapedesis, although CXCL8 concentrations were high. Generally, CXCL8 has a crucial, but not exclusive role in neutrophil recruitment [50]. Different combinations of locally produced cytokines can manipulate the subsequent inflammatory responses. For example, IL-17A and IFN-γ are known to reciprocally regulate one another [51]. IFN-γ has previously been reported to inhibit neutrophil accumulation in the lung during Mtb infection, suggesting that this cytokine could possess anti-inflammatory properties [52]. Another cytokine that affects neutrophil recruitment is IL-17 that induces the release of pro-inflammatory factors such as cytokines, chemokines and MMPs) from mesenchymal and myeloid cells, leading to recruitment and activation of neutrophils [25]. In contrast to previous studies, we did not find neutrophil/epithelial MMP-1/IL-17 production upon BCG stimulation [53, 54]. However, addition of IL-17A during BCG infection increased epithelial IL-6 secretion and sustained BCG-induced neutrophil diapedesis. IL-17A is further known to mediate vaccine-induced immunity by binding to its homodimeric or heterodimeric receptors, such as IL-17RA/IL-17RB [55]. We observed that primary epithelial cells and neutrophils expressed the IL-17RA. This receptor forms dimers with IL-17RA, IL-17RB, IL-17RC or IL-17RE in order to bind several members of the IL-17 cytokine family [27]. We found also that primary AECs expressed IL-17RE, suggesting the epithelial IL-17RA/RE dimer could be binding IL-17C. Epithelial IL-17C was previously observed to act in an autocrine manner to sustain the inflammatory response of the mucosa [27], but we did not find epithelial or neutrophil IL-17C or IL-17A production upon BCG stimulation. Interestingly, neutrophil IL-17RA and epithelial IL-17RE expression decreased upon BCG stimulation, while epithelial IL-17RA expression remained steadily expressed. Decreased receptor expression could control IL-17 signalling as these pathways activate transcription factors AP-1, NF-kB and C/EBP [55].

There are several opportunities for the improvement of the current vaccine for TB. Our study reveals that early events after BCG vaccination lead to mucosal and neutrophil activation that could affect the protective qualities against pulmonary tuberculosis. However for future vaccine strategies, the immune response could be improved to increase the local production of IL-6 and IL-17 cytokines in order to enhance vaccine immunogenicity.

## Supporting Information

S1 Fig Flow cytometry optimization for IL-17RE. Airway epithelial cells were harvested and the incubated with primary antibodies against IL-17RE (unstimulated), unspecific IgG control (IgG) at a concentrations ranging from 1–10 μg/ml. Median fluorescence intensity (MFI) was compared between the two groups and background auto-fluorescence (Background).
(TIF)

S2 Fig Neutrophil migration by addition of IL-1α or CXCL-8. Epithelial cells were seeded to the bottom compartment of a transwell system. (A) IL-1α was added to the bottom compartment for 24 hours. (B) CXCL-8 was added to the uninfected epithelial cells and neutralizing antibodies against CXCL-8 was added to BCG infected epithelial cells. Primary neutrophils were added to a layer of HUVEC cells in the top insert well. Neutrophil transmigration over the membrane was measured by counting the number of neutrophils in the bottom well compared to the insert after 3 hours. Result is depicted as mean ± SEM percentage of neutrophil diapedesis. Means of samples were compared with medium control using ANOVA followed by Dunnet’s multiple comparisons test and significance was accepted at *p < 0.05, **p< 0.01, or ***p < 0.001.
(TIF)

S3 Fig Neutrophil expression of FOXO3a and pFOXO3a during BCG infection. Primary neutrophils were stimulated with BCG for one and two hours in the presence of IL-17A and/or IFN-γ. FOXO3 levels were determined by Western blot. Results are depicted as mean ± SD normalized to GAPDH (n = 3).
(TIF)

S4 Fig Neutrophil apoptosis by Annexin V assay. Primary neutrophils were stimulated with BCG for two hours. Apoptosis was compared between infected (black) or uninfected (gray) cells by Annexin V. Results are shown as representative histograms for each condition.
(TIF)
