# Supplementary figures and images for: Innate Immune Responses after Airway Epithelial Stimulation with Mycobacterium bovis Bacille-Calmette Guérin
Source: PLoS One. 2016 Oct 10;11(10):e0164431. doi: 10.1371/journal.pone.0164431 (PMC5056730; doi:10.1371/journal.pone.0164431)

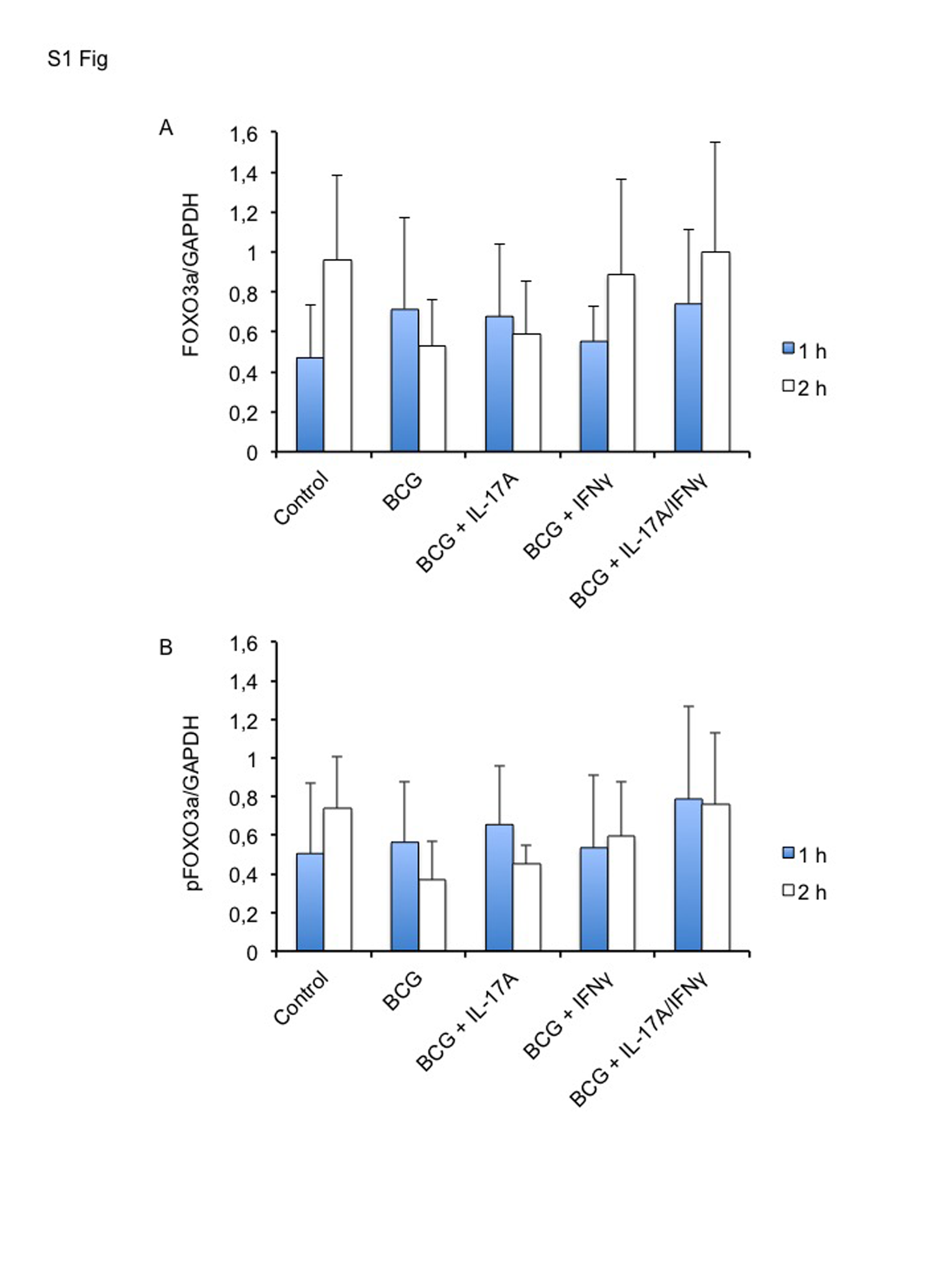

Supplement: S1 Fig — Airway epithelial cells were harvested and the incubated with primary antibodies against IL-17RE (unstimulated), unspecific IgG control (IgG) at a concentrations ranging from 1–10 μg/ml. Median fluorescence intensity (MFI) was compared between the two groups and background auto-fluorescence (Background). (TIF) [file pone.0164431.s001.tif]

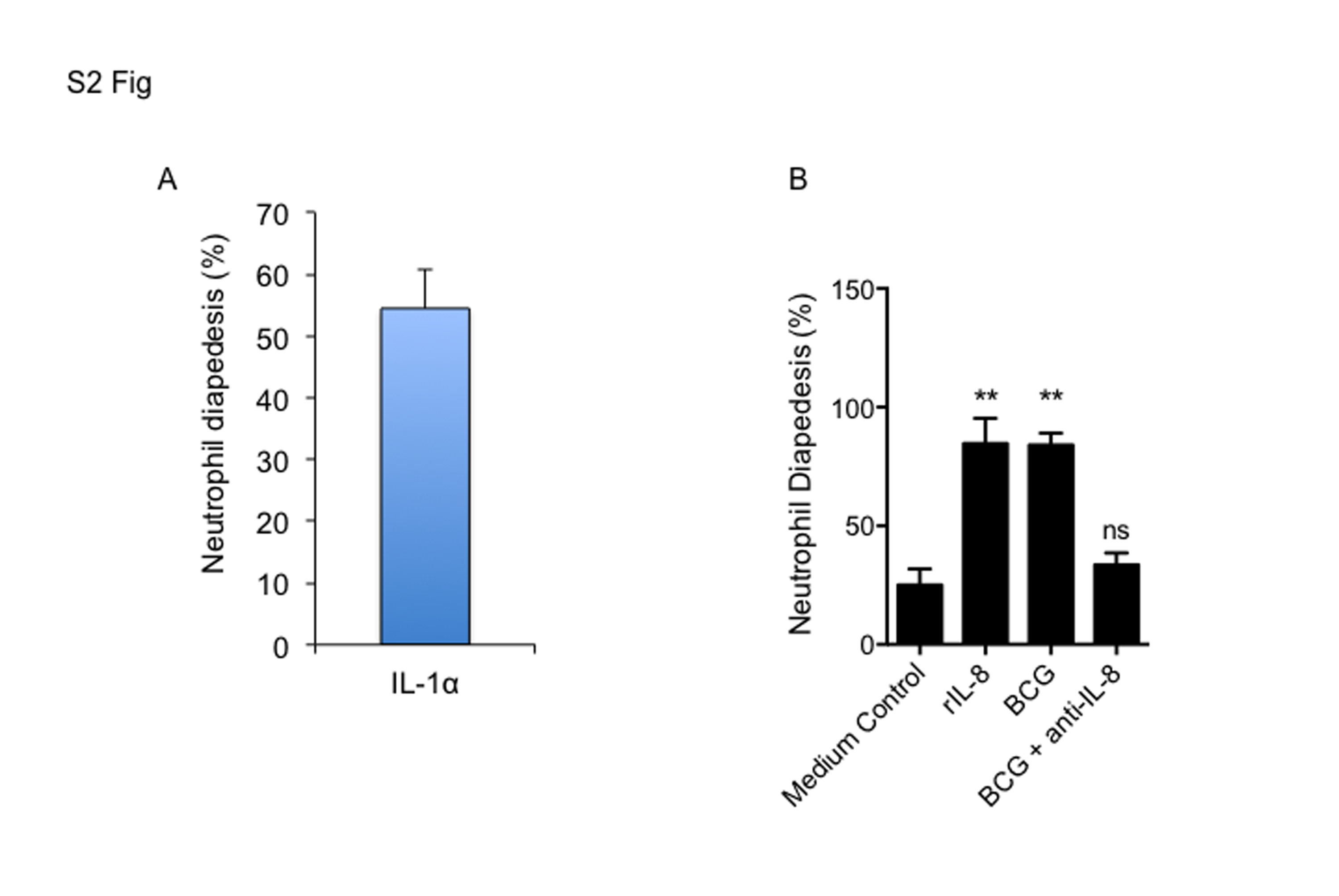

Supplement: S2 Fig — Epithelial cells were seeded to the bottom compartment of a transwell system. (A) IL-1α was added to the bottom compartment for 24 hours. (B) CXCL-8 was added to the uninfected epithelial cells and neutralizing antibodies against CXCL-8 was added to BCG infected epithelial cells. Primary neutrophils were added to a layer of HUVEC cells in the top insert well. Neutrophil transmigration over the membrane was measured by counting the number of neutrophils in the bottom well compared to the insert after 3 hours. Result is depicted as mean ± SEM percentage of neutrophil diapedesis. Means of samples were compared with medium control using ANOVA followed by Dunnet’s multiple comparisons test and significance was accepted at *p < 0.05, **p< 0.01, or ***p < 0.001. (TIF) [file pone.0164431.s002.tif]

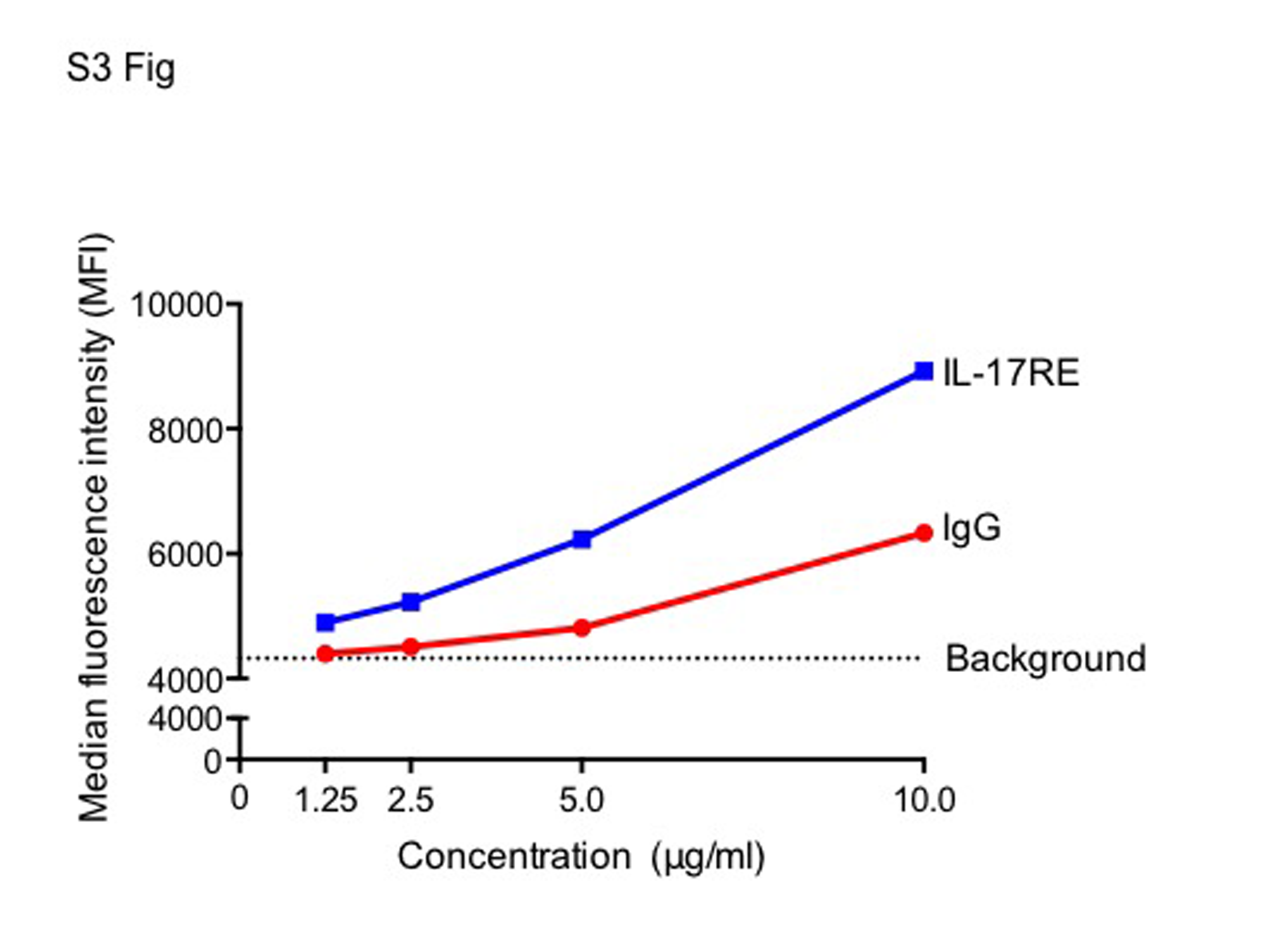

Supplement: S3 Fig — Primary neutrophils were stimulated with BCG for one and two hours in the presence of IL-17A and/or IFN-γ. FOXO3 levels were determined by Western blot. Results are depicted as mean ± SD normalized to GAPDH (n = 3). (TIF) [file pone.0164431.s003.tif]

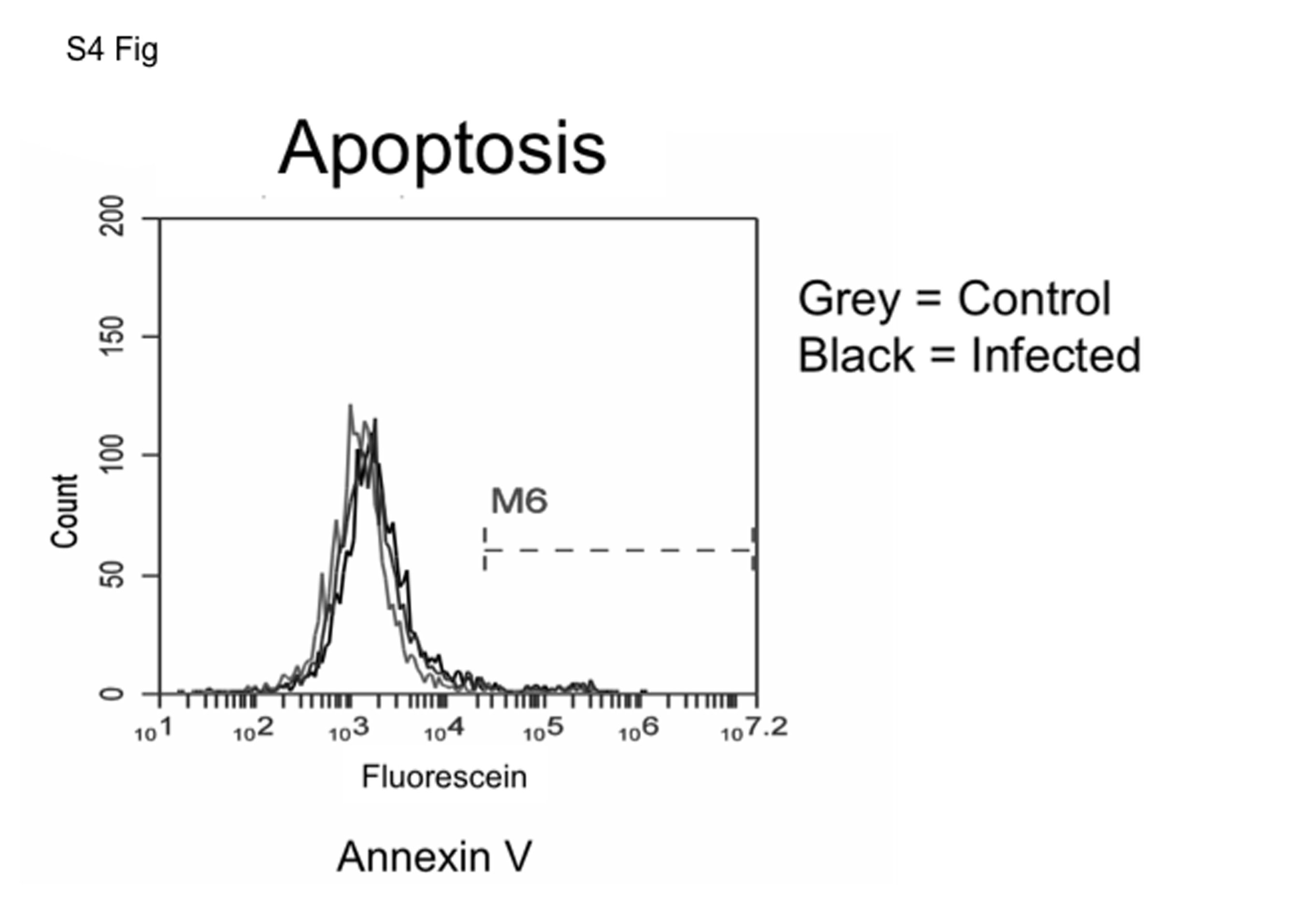

Supplement: S4 Fig — Primary neutrophils were stimulated with BCG for two hours. Apoptosis was compared between infected (black) or uninfected (gray) cells by Annexin V. Results are shown as representative histograms for each condition. (TIF) [file pone.0164431.s004.tif]
